# Kiloh-Nevin Syndrome

**DOI:** 10.5334/jbsr.2183

**Published:** 2020-09-11

**Authors:** Victor Rabaut, Vincent VandeVyver, Koenraad Verstraete

**Affiliations:** 2 Ghent University, BE; 2 AZ Alma, BE

**Keywords:** Kiloh-Nevin Syndrome, entrapment, rare, forearm, MRI, neuropathy, median nerve

## Abstract

**Teaching point:** The Kiloh-Nevin Syndrome is a rare entrapment syndrome of the median nerve, with a distinct muscle edema pattern of the forearm.

## Case

A 50-year-old man was referred for radiological investigation because he had difficulties with flexing the interphalangeal joint of the left thumb and the distal interphalangeal joint of the left index without any pain or other sensory issues. There was no specific direct trauma prior to the complaints.

**Figure 1:**
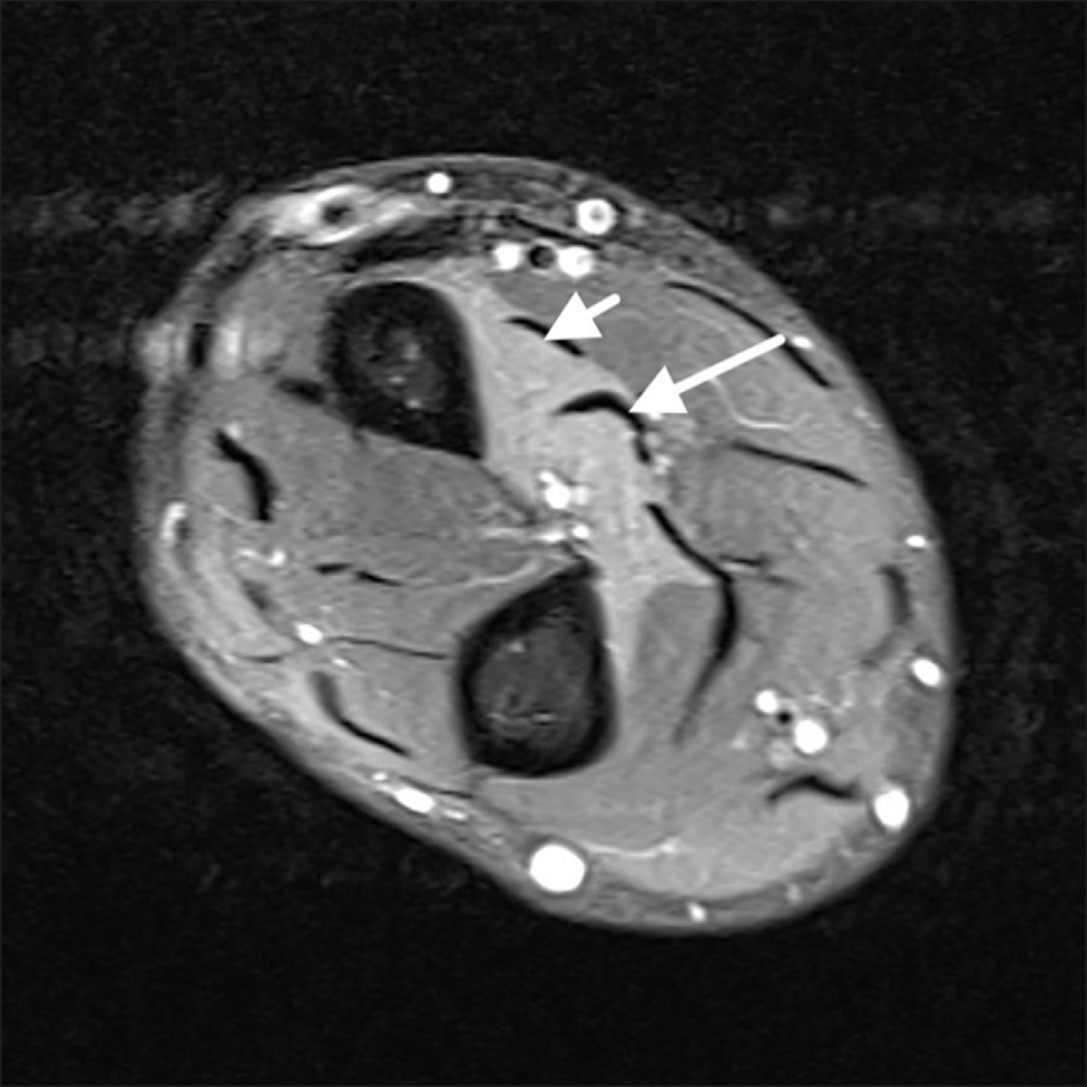
Fat saturated intermediate-weighted proximal axial view.

**Figure 2:**
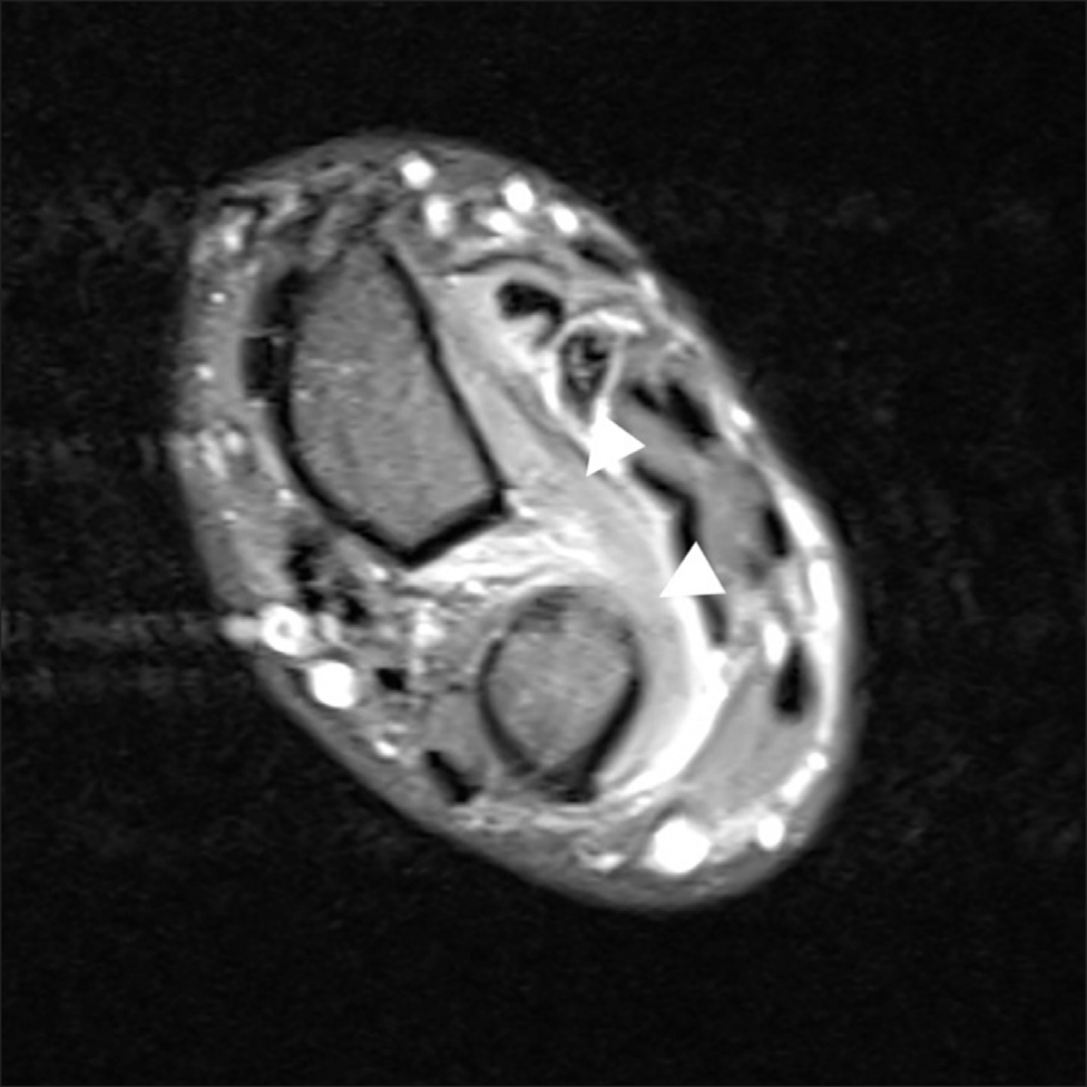
Fat saturated intermediate-weighted distal axial view.

First, the patient underwent an ultrasound and magnetic resonance imaging (MRI) of his left hand. These ruled out tendinopathy. Furthermore, the patient underwent MRI of the cervical spine, which did not detect any cervical neuropathy. Clinically, the inability to perform the ‘OK sign’ was described. MRI of the left forearm was carried out with axial T1WI and fat saturated intermediate-weighted images. Fat-saturated intermediate-weighted images showed homogeneous increased signal intensity of the flexor pollicis longus (short arrow, Figure 1), the flexor digitorum profundus (long arrow, Figure 1) and the pronator quadratus (arrowhead, Figure 2), compatible with denervation edema. There was no fatty infiltration on T1WI nor muscle atrophy, which indicated an early phase of the disease. The pattern of muscle edema was highly suggestive for Kiloh-Nevin syndrome. The findings were supported by a specifically conducted electromyogram (EMG). The patient was initially treated conservatively.

## Comment

Kiloh-Nevin syndrome or anterior interosseous nerve syndrome (AINS) is a rare entrapment syndrome of a branch of the median nerve. It is a motor neuropathy, affecting the flexor pollicis longus, flexor digitorum profundus and pronator quadratus. Clinically, the patient fails to make an ‘OK sign’. Due to compression of the nerve, flexion of the interphalangeal joint of the thumb and the distal interphalangeal joint of the index is impaired and the pinching maneuver disappears. AINS can be diagnosed with EMG or imaging techniques. The most indicated imaging modality is MRI, which can detect pathological muscle signal changes before neurologic changes are detectable on EMG. In the acute phase, short-tau inversion-recovery, and fat saturated T2- or intermediate-weighted sequences show a diffuse, homogeneous increased signal intensity of the muscles without affecting fat or fascia, also known as denervation edema. It must be noted that only flexion of the thumb and index is impaired, so only the corresponding radial part of the flexor digitorum profundus will be affected. In more chronic stages, T1-weighted sequences will show fatty infiltration and amyotrophy. Etiologically, the site of impingement is rarely identified on MRI. Most commonly the anterior interosseous nerve is compressed by the tendinous origin of the deep head of the pronator teres. Rarely, a mass or inflammation is seen in course of the nerve to explain the symptoms. Traumatic nerve damage can be caused by surgery, venous puncture, or cast pressure. The Kiloh-Nevin syndrome can be treated conservatively with NSAIDs and corticosteroids. For severe and refractory cases, decompression surgery is needed [1].

## References

[1] Andreisek G, Crook DW, Burg D, Marincek B, Weishaupt D. Peripheral neuropathies of the median, radial, and ulnar nerves: MR imaging features. RadioGraphics. 2006; 26: 1267–1287. DOI: 10.1148/rg.265055712 16973765

